# Linkages Between Nutrient Resorption and Ecological Stoichiometry and Homeostasis Along a Chronosequence of Mongolian Pine Plantations

**DOI:** 10.3389/fpls.2021.692683

**Published:** 2021-08-13

**Authors:** Kai Wang, G. Geoff Wang, Lining Song, Risheng Zhang, Tao Yan, Yihang Li

**Affiliations:** ^1^College of Environmental Sciences and Engineering, Liaoning Technical University, Fuxin, China; ^2^Department of Forestry and Environmental Conservation, Clemson University, Clemson, SC, United States; ^3^Institute of Applied Ecology, Chinese Academy of Sciences, Shenyang, China; ^4^Department of Desertification Control, Liaoning Institute of Sandy Land Control and Utilization, Fuxin, China; ^5^College of Pastoral Agriculture Science and Technology, Lanzhou University, Lanzhou, China

**Keywords:** nutrient conservation strategy, nutrient limitation, stand development, stoichiometric homeostasis, *Pinus sylvestris* var *mongolica*

## Abstract

Nutrient resorption is an important strategy for nutrient conservation, particularly under conditions of nutrient limitation. However, changes in nutrient resorption efficiency with stand development and the associated correlations with ecological stoichiometry and homeostasis are poorly understood. In the study, the authors measured carbon (C), nitrogen (N), and phosphorus (P) concentrations in soil and in green and senesced needles along a chronosequence of Mongolian pine (*Pinus sylvestris* var. *mongolica*) plantations (12-, 22-, 31-, 42-, 52-, and 59-year-old) in Horqin Sandy Land of China, calculated N and P resorption efficiency (NRE and PRE, respectively), and homeostasis coefficient. The authors found that soil organic C and total N concentrations increased, but soil total P and available P concentrations decreased with stand age. Green needle N concentrations and N:P ratios as well as senesced needle C:N ratios, NRE, and PRE exhibited patterns of initial increase and subsequent decline with stand age, whereas green needle C:N ratios and senesced needle N concentrations, and N:P ratios exhibited the opposite pattern. NRE was positively correlated with N concentration and N:P ratio, but negatively correlated with C:N ratio in green needles, whereas the opposite pattern was observed in senesced needles. PRE was negatively correlated with senesced needle P concentration, soil-available N concentration, and available N:P ratio. The homeostatic coefficient of N:P was greater when including all stand ages than when including only those younger than 42 years. These findings indicate that tree growth may change from tending to be N limited to tending to be P limited along the Mongolian pine plantation chronosequence. Nutrient resorption was coupled strongly to tree growth and development, whereas it played a lesser role in maintaining stoichiometric homeostasis across the plantation chronosequence. Therefore, adaptive fertilization management strategies should be applied for the sustainable development of Mongolian pine plantations.

## Introduction

Nitrogen (N) and phosphorus (P) play important roles in plant growth and metabolism, and deficiencies thereof have been shown to strongly limit forest growth and productivity (Vitousek et al., 2010; See et al., 2015). During forest stand development, plant photosynthetic characteristics, nutrition requirements, and soil nutrient supply often remain changed (Zhang et al., 2018). These variations in soil and plant nutrient status may cause a transition in the type of nutrient limitation; N limitation often occurs in young forests, whereas P limitation tends to progressively occur in aging forests, particularly in areas with nutrient deficiencies (Yan et al., 2018; Deng et al., 2019). Such changes in nutrient limitation status across a forest chronosequence would have substantial effects on plant survival and growth. Understanding these effects will enhance our appreciation of the adaptability of plants to changes in nutrients in an environment.

Ecological stoichiometry, which studies the balance of energy and chemical elements in ecological interactions (Elser et al., 2010), offers an option of investigating the changes in multiple elements during the growth and development of forest ecosystems (Yan et al., 2018). Carbon (C):N:P stoichiometry has been used to explore the relationships and feedback between plants and soil in ecological processes (Wang et al., 2007). Leaf N:P ratios are widely used to indicate N (N:P < 14) and/or P (N:P > 16) limitation for the growth of plants (Koerselman and Meuleman, 1996). Soil C:N, C:P, and N:P ratios not only reflect soil fertility and nutrient limitation, but also affect plant nutrient state and regulate plant growth (Fan et al., 2015). Stoichiometric homeostasis, the central concept of ecological stoichiometry, is defined as the ability of an organism to maintain a relatively constant nutrient composition regardless of changes in environmental conditions (Sterner and Elser, 2002). Stoichiometric homeostasis has been validated in trees (Wang et al., 2019), shrubs (Gu et al., 2017), and herbs (Yu et al., 2011). Stoichiometric homeostasis may have different limits in response to changing environments (Meunier et al., 2014), while the degree of homeostasis also varies during different growth stages in plants (Peng et al., 2016).

Nutrient resorption from senescing organs is an important mechanism by which plants conserve nutrients and optimize their use efficiency (Aerts, 1996), making them less dependent on the soil nutrient supply and helps to maintain stoichiometric homeostasis (Brant and Chen, 2015). Plants are expected to have greater N or P resorption efficiency (NRE or PRE, respectively) under N- or P-limited conditions (Killingbeck, 1996; Yan et al., 2018). NRE:PRE ratio is used as an indicator of nutrient limitation, considering that plants should use N or P more efficiently relative to the other nutrient under selective pressure (Reed et al., 2012). Aside from the impact of soil nutrient status, nutrient resorption efficiency (NuRE) can also be affected by leaf and litter nutrients (Kobe et al., 2005; Deng et al., 2019), leading to various patterns in response to stand age, such as an increase (Ye et al., 2012; Yan et al., 2018), decrease (Li et al., 2013), or no significant change (Zhang et al., 2018). It is commonly accepted that NuRE is negatively correlated with soil nutrients (Tully et al., 2013). However, some studies suggested that plants growing on lower fertility soil did not always have higher NuRE (Aerts, 1996), and fertilization study also showed various responses of NuRE to nutrient addition (Chen et al., 2015; Deng et al., 2016; Li et al., 2016). Nutrient resorption is a key mechanism in maintaining homeostasis. The higher the NuRE is, the higher homeostasis and stable nutrient composition plants usually have (Julian et al., 2020). However, some studies suggested contrasting nutrient homeostasis and resorption responses to environmental nutrient availability across growth stages (Peng et al., 2016). Accordingly, as the variations in NuRE with stand development and the associated correlations with stoichiometry and homeostasis remain unclear, it is necessary to link nutrient resorption to stoichiometry and homeostasis to reveal the nutrient conservation mechanisms across a forest chronosequence.

Mongolian pine (*Pinus sylvestris* var. *mongolica*) is the dominant afforestation species in “Three Norths” area of China (northwest, north, and northeast China; Zhu et al., 2006), and its afforestation area has reached more than 7.0 × 10^5^ ha (Song et al., 2019). However, the earliest Mongolian pine plantations to be introduced have begun to decline, as indicated by observed crown dieback, lack of natural regeneration, low growth rate, and high mortality (Zhu et al., 2006). Water supply and nutrient availability are the main limiting factors for plant growth in the sandy land (Zhao et al., 2009; Song et al., 2019). Nutrient limitation often occurs in pure plantations due to the single-species composition and mono-silviculture system in arid and semiarid areas (Deng et al., 2019). Several studies have revealed N limitation for the growth of Mongolian pine (Chen et al., 2004), whereas others have demonstrated soil P deficiency in the plantations (Zhao et al., 2009). The disagreements may be because earlier studies were conducted only at one specific stage of growth or with a narrow range of stand ages of Mongolian pine (Zhao et al., 2009). Thus, nutrient limitation status during the development of Mongolian pine plantations remains unclear. Knowledge of nutrient resorption changes with stand age and the feedback between tree nutrient conservation strategies and soil nutrient availability would help gain a better understanding of soil nutrient limitation, control, and consequences (Yan et al., 2018). Therefore, linking nutrient resorption to stoichiometry and homeostasis across a forest chronosequence would have significant implications to understand the mechanisms of decline and proper management of Mongolian pine plantations.

The purpose of this study was to understand how nutrient resorption changes with stand development and how it associates with stoichiometry for Mongolian pine plantations. We examined the patterns of soil and needle C, N, and P stoichiometry and needle nutrient resorption in Mongolian pine plantations across a chronosequence consisting of 12-, 22-, 31-, 42-, 52-, and 59-year-old stands in the Horqin Sandy Land of China. The study also analyzed the relationships between nutrient resorption and stoichiometry and homeostasis. We hypothesized the following (i) NuRE would increase along the plantation chronosequence since nutrient demand may increase as stands developed and (ii) NuRE would be negatively related to soil and needle nutrient status since nutrient resorption is important to conserve nutrients for plants.

## Materials and Methods

### Study Site

This study was conducted at the Zhanggutai Experimental Base of the Liaoning Institute of Sandy Land Control and Utilization, Liaoning Province, China (42°43′ N, 122°22′ E), which is located in the southeastern region of the Horqin Sandy Land (Supplementary Figure 1). The Zhanggutai experimental base covers an area of 2,620 hm^2^, with Mongolian pine plantation accounting for 1,587 hm^2^. Mongolian pine was first introduced for afforestation in the region. Thus, it includes the oldest plantations. The region has a semiarid climate and the average altitude is 226 m above mean sea level. The mean annual temperature is approximately 7.7°C, with minimum and maximum air temperatures of −29.5 and 37.2°C, respectively. The mean annual precipitation is 474 mm, and the mean annual potential pan evaporation is ~1,580 mm (Song et al., 2019). According to the FAO soil classification (Food Agriculture Organization of the United Nations, 2015), the soil type is arenosol, which develops from sandy parent material through wind action (Zhu et al., 2006). The vegetation mainly consists of psammophytes, which belong to the flora of Inner Mongolia. A large area of shelterbelts included *P. sylvestris* var. *mongolica, Pinus tabuliformis, Populus*. spp., and *Ulmus pumila* that were planted in the region for sand fixation and agricultural production since the 1950s.

### Experimental Design

In August 2017, we selected 12-, 22-, 31-, 42-, 52-, and 59-year-old pure Mongolian pine plantation stands at the Zhanggutai experimental base to form a chronosequence. All plantations were established from non-vegetated sandy lands. Previous studies have shown that soil properties were relatively similar prior to afforestation, and that the C, N, and P concentrations were 3.15, 0.24, and 0.09 g kg^−1^, respectively (Chen et al., 2004; Zhang et al., 2013; Wang et al., 2021b). The initial afforestation density was 2 × 3 m for all the stands. Although the foliar litter was occasionally collected away by the local people more than a decade ago, no one collects foliar litter in recent years. No fertilization management was conducted in any of the stands. The 12-, 22-, 31-, 42-, 52-, and 59-year-old stands were thinned by 25% in 2014, 25% in 2002, 30% in 2015, 25% in 2002 and 2011; 25% in 2002, 30% in 2015, and 25% in 1995, 2002, and 2015. Three replicated stands were sampled in each age class, and a 20 × 20 m plot was established within each stand. All plots were on flat topography and within 10 km, with similar soil type (arenosols), elevation, and climatic condition (Table 1). Tree height, diameter at breast height (DBH), and site conditions were recorded for all the stands. Within each plot, three healthy individuals (without diseases and/or insect pests) with average DBH were randomly selected, and the distance between any two sample trees was >5 m.

**Table 1:** Characteristics of the Mongolian pine plantations with different ages.

**Stand age (year)**	**Year of afforestation**	**Elevation (m)**	**Density (trees ha^**−1**^)**	**Mean height (m)**	**Mean DBH (cm)**	**Canopy density**	**Soil water content (%)**
12	2007	230.4 ± 15.2	1, 205 ± 16	2.32 ± 0.08	3.54 ± 0.22		5.26 ± 0.10
22	1997	253.4 ± 15.9	1, 011 ± 14	3.92 ± 0.10	9.42 ± 0.36	0.54 ± 0.03	7.19 ± 0.18
31	1988	208.3 ± 14.1	708 ± 7	8.13 ± 0.18	15.35 ± 0.48	0.74 ± 0.04	6.21 ± 0.26
42	1977	230.4 ± 12.1	310 ± 7	9.54 ± 0.26	19.95 ± 0.61	0.66 ± 0.02	4.74 ± 0.31
52	1967	252.5 ± 14.2	291 ± 7	12.02 ± 0.43	23.21 ± 0.52	0.45 ± 0.02	5.42 ± 0.47
59	1960	233.5 ± 7.9	270 ± 6	13.25 ± 0.39	23.52 ± 0.66	0.45 ± 0.03	4.73 ± 0.03

*DBH, diameter at breast height*.

Green and senesced needles from the same tree were sampled in mid-August and October, when the needles were at peak biomass and maximum abscission, respectively. Twelve branches from lower to upper and from four opposite compass sides of the crown of each individual were cut away using a tree trimmer or by climbing the trees. All age-class green needles without diseases and/or insect pests were collected from these branches and mixed. Additionally, senesced needles were collected directly from the branch of trees rather than from litter to avoid underestimating their nutrient concentrations (Li et al., 2013). The needles were completely yellow, dry, and ready to fall off if the branch was given a gentle shake or touched (Yan et al., 2016, 2018). For tall trees, the branches were cut away and then collected senesced needles. All needle samples were dried for 72 h at 60°C, ground with a mechanical grinder, passed through a 0.149-mm sieve, and then used to measure total C, N, and P concentrations.

As more than 60% of fine roots were distributed within the 0–40 cm soil layer (Wang et al., 2014), soil samples from depths of 0–10, 10–20, and 20–40 cm were taken when sampling green needles. After removing the understory plants and surface litter, four soil samples were randomly collected within 1 m of the base of each selected tree using a metal tube (5 cm in diameter) and pooled to obtain one composite soil sample per tree (Yan et al., 2018). Roots and stones were removed from the samples. Some soil samples were air-dried and passed through a 0.149-mm mesh for organic C, total N, and total P concentration analysis. The remaining fresh soil samples were passed through a 1-mm mesh and then used to measure available N and P concentrations.

### Chemical Measurements

Total needle C and soil organic C concentrations were measured using the oil bath-K_2_Cr_2_O_7_ titration method. Total N concentrations in the needles and soil were determined according to the semimicro-Kjeldahl method using a Kjeldahl autoanalyzer (JY-SPD60, Beijing, China). Total P concentrations in the needles and soil were determined according to the colorimetric method using a spectrophotometer (T6, Beijing, China) after digestion in H_2_SO_4_-H_2_O_2_ and H_2_SO_4_-HClO_4_, respectively. Soil-available N concentrations were analyzed using the alkali diffusion method. Soil-available P concentrations were measured according to the colorimetric method using a spectrophotometer after extraction with NaHCO_3_ (Bao, 2000).

### Calculations

Nutrient resorption efficiency was used to quantify nutrient resorption, which was calculated as follows (Vergutz et al., 2012):

$$\begin{array}{l} NuRE=\frac{N_{g}\;-\;N_{s}\;\times \;MLCF}{N_{g}}\;\times \;100\%, \end{array}$$

where the mass loss correction factor value was 0.745 for conifers (Vergutz et al., 2012), and *N*_g_ and *N*_s_ represented the nutrient concentrations in all age-class green needles and senesced needles, respectively.

The homeostatic coefficient (*H*) was calculated using the nutrient concentrations and their ratios in needles and soil. The *H*-value was derived from the following model (Sterner and Elser, 2002):

$$\begin{array}{l} y=c\;+\;\frac{1}{H}\;\times \;\log x, \end{array}$$

where *y* is the N concentration, P concentration, or N:P ratio in the needles, and *x* is the corresponding value in the soil. The total nutrient concentrations and their ratios at 0–40 cm were used as soil values, which were calculated as the means of the different soil layers. *H* and *c* were obtained through a linear regression analysis. If the regression relationship was non-significant (*P* > 0.05), the slope (1/*H*) was set to 0. If the regression relationship was significant (*P* < 0.05), a slope of 0 indicates strict homeostasis, and that foliar nutrient stoichiometry remained unchanged with soil nutrient stoichiometry. A slope between 0 and 1 indicates homeostatic adjustment of foliar nutrient stoichiometry and organism nutrient accumulation. A slope equal to 1 indicates non-homeostasis and the foliar nutrient stoichiometry reflected soil nutrient stoichiometry. A slope >1 indicates that the organism accumulated nutrients at a faster rate than the rate of increase in nutrients in the soil (Meunier et al., 2014).

### Statistical Analyses

Needle and soil C, N, and P stoichiometric ratios were calculated as mass ratios. The normality of data was checked using the Kolmogorov–Smirnov test, and the homogeneity of variance was examined using Levene's test. Subsequently one-way analysis of variance was used to compare the significant differences in the soil nutrients and NuREs among the different-aged plantations and soil nutrients among the different soil layers. Duncan's test was conducted for *post-hoc* multiple comparisons. Differences between NRE and PRE within stands of the same age were analyzed using the two-sample *t*-test. Linear and quadratic regression analyses were performed to test the relationships between stand age and needle nutrient concentrations and their ratios, and NRE:PRE ratios, and the relationship was determined by which model was more statistically significant. Analysis of covariance was used to test for significant differences in slopes of nutrient stoichiometry between green and senesced needles, and slopes of stoichiometric homeostasis between stands of all ages and those younger than 42 years. Pearson's bivariate correlations were used to determine the relationships between NuRE and needle and soil nutrient concentrations and their ratios, and between soil-available N:P ratios and soil total N:P and needle N:P ratios. All statistical analyses were performed using SPSS 16.0 (SPSS Inc., Chicago, IL, USA) for Windows. Statistical significance was set at *P* < 0.05.

## Results

### Soil C, N, and P Concentrations and Stoichiometric Ratios

Soil organic C concentrations tended to increase with stand age at all observed soil layers (Table 2). Soil total N concentrations exhibited an upward trend at 0–10 cm, and the highest concentrations were observed in the 22-year-old stands in the 10–20 and 20–40 cm soil layers (Table 2). Soil total and available P concentrations tended to decrease with stand age. However, soil-available N concentrations increased and then declined, with the greatest values being found in the 22-year-old stands (Table 2). Generally, total C:P and N:P ratios, and available N:P ratios showed upward trends with stand age (Table 2).

**Table 2:** Soil nutrient concentrations along the stand age chronosequence of Mongolian pine plantations.

**Soil nutrient**	**Soil layer (cm)**	**Stand age (year)**					
		**12**	**22**	**31**	**42**	**52**	**59**
Organic C (g kg^−1^)	0–10	3.27 ± 0.13c	3.39 ± 0.13c	3.31 ± 0.12c	5.14 ± 0.17b	5.36 ± 0.09b	6.02 ± 0.12a
	10–20	1.94 ± 0.09c	2.93 ± 0.05a	2.50 ± 0.11b	2.66 ± 0.12ab	2.96 ± 0.17a	2.83 ± 0.10ab
	20–40	1.78 ± 0.09d	2.47 ± 0.10b	2.21 ± 0.09bc	2.03 ± 0.04cd	2.52 ± 0.03b	3.27 ± 0.18a
Total N (g kg^−1^)	0–10	0.36 ± 0.02c	0.45 ± 0.01b	0.42 ± 0.02b	0.55 ± 0.02a	0.54 ± 0.02a	0.57 ± 0.02a
	10–20	0.21 ± 0.01c	0.36 ± 0.01a	0.30 ± 0.02b	0.32 ± 0.01ab	0.30 ± 0.01b	0.35 ± 0.01a
	20–40	0.24 ± 0.01bc	0.29 ± 0.01a	0.22 ± 0.01c	0.25 ± 0.01abc	0.24 ± 0.02bc	0.28 ± 0.01ab
Total P (g kg^−1^)	0–10	0.118 ± 0.005a	0.120 ± 0.005a	0.108 ± 0.006ab	0.106 ± 0.002ab	0.097 ± 0.002b	0.082 ± 0.004c
	10–20	0.108 ± 0.005a	0.095 ± 0.003b	0.110 ± 0.004a	0.111 ± 0.005a	0.090 ± 0.001bc	0.081 ± 0.002c
	20–40	0.107 ± 0.005ab	0.113 ± 0.004a	0.099 ± 0.002b	0.084 ± 0.006c	0.078 ± 0.004c	0.080 ± 0.001c
C:N	0–10	9.02 ± 0.17b	7.56 ± 0.11c	7.84 ± 0.14c	9.43 ± 0.14b	10.01 ± 0.23a	10.48 ± 0.12a
	10–20	9.16 ± 0.13b	8.26 ± 0.14c	8.32 ± 0.25c	8.23 ± 0.21c	9.99 ± 0.27a	8.03 ± 0.03c
	20–40	7.47 ± 0.22c	8.55 ± 0.19c	10.26 ± 0.44b	8.03 ± 0.31c	10.66 ± 0.63b	11.82 ± 0.19a
C:P	0–10	27.76 ± 0.23d	28.33 ± 0.69d	30.92 ± 0.64d	48.78 ± 0.74c	55.11 ± 1.56b	73.73 ± 2.14a
	10–20	17.94 ± 0.51d	30.77 ± 0.43b	22.74 ± 0.43c	24.07 ± 0.28c	33.02 ± 1.61ab	35.00 ± 0.39a
	20–40	16.59 ± 0.12d	21.96 ± 0.17c	22.30 ± 0.50c	24.27 ± 1.12c	32.41 ± 0.99b	40.68 ± 1.86a
N:P	0–10	3.08 ± 0.07d	3.75 ± 0.05c	3.95 ± 0.04c	5.18 ± 0.12b	5.52 ± 0.28b	7.04 ± 0.15a
	10–20	1.96 ± 0.04e	3.73 ± 0.03b	2.74 ± 0.10d	2.93 ± 0.08d	3.30 ± 0.08c	4.37 ± 0.03a
	20–40	2.23 ± 0.06d	2.57 ± 0.04c	2.18 ± 0.11d	3.02 ± 0.03b	3.05 ± 0.11b	3.44 ± 0.11a
Available N (mg kg^−1^)	0–10	37.50 ± 1.90d	80.08 ± 2.16a	61.79 ± 3.08b	50.14 ± 2.29c	53.79 ± 1.82c	53.31 ± 2.00c
	10–20	28.41 ± 0.29c	56.40 ± 2.16a	41.27 ± 1.96b	31.88 ± 0.75c	31.55 ± 1.15c	32.91 ± 1.72c
	20–40	25.34 ± 0.44c	48.61 ± 2.26a	36.51 ± 1.26b	27.95 ± 1.57c	25.98 ± 0.69c	25.52 ± 1.32c
Available P (mg kg^−1^)	0–10	7.58 ± 0.26a	7.24 ± 0.24a	6.00 ± 0.08b	4.65 ± 0.14c	3.87 ± 0.08d	3.54 ± 0.17d
	10–20	6.25 ± 0.25c	8.14 ± 0.09a	7.27 ± 0.07b	6.50 ± 0.18c	3.82 ± 0.13d	3.40 ± 0.13d
	20–40	7.20 ± 0.12a	6.90 ± 0.07ab	6.66 ± 0.13b	4.68 ± 0.06c	3.73 ± 0.21d	3.53 ± 0.10d
Available N:P	0–10	4.93 ± 015e	10.96 ± 0.09c	10.00 ± 0.32d	10.76 ± 0.18c	13.90 ± 0.20b	14.98 ± 0.31a
	10–20	4.53 ± 0.04e	7.06 ± 0.23c	5.58 ± 0.25d	4.80 ± 0.04e	8.40 ± 0.12b	9.40 ± 0.15a
	20–40	3.44 ± 0.03c	6.94 ± 0.15a	5.43 ± 0.13b	5.88 ± 0.28b	7.05 ± 0.14a	7.29 ± 0.19a

*Different lowercases within the same line indicate significant differences among stand ages (P < 0.05). Values are the mean ± SE (n = 3)*.

### Needle C, N, and P Concentrations and Stoichiometric Ratios

**Figure 1:**
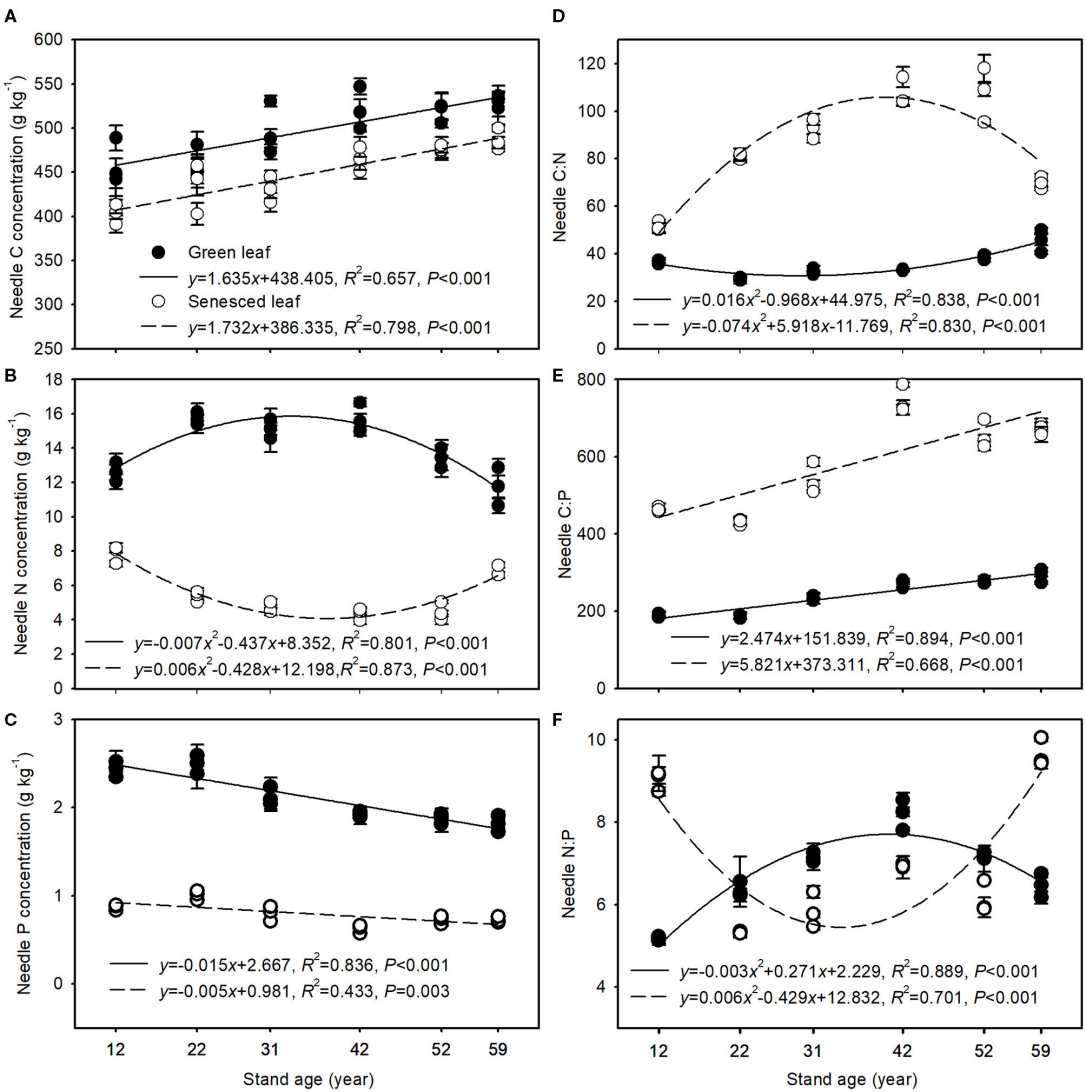
Changes in C **(A)**, N **(B)**, and P **(C)** concentrations and C:N **(D)**, C:P **(E)**, and N:P **(F)** in green and senesced needles along the chronosequence of Mongolian pine plantations. Solid and dashed lines indicate the regression equation for green and senesced needles, respectively. Data are shown as the mean ± SE (*n* = 3).

Both green and senesced needle C concentrations increased significantly with stand age, ranging from 459.78 to 529.79 g kg^−1^ and from 403.04 to 486.65 g kg^−1^, respectively (Figure 1A). By contrast, green needle N concentrations tended to rise and subsequently drop, whereas senesced needle N concentrations tended to decrease and then increase with stand age (Figure 1B). However, green and senesced needle P concentrations decreased significantly with stand age, with values ranging from 2.49 to 1.81 and 1.01 to 0.72 g kg^−1^, respectively (Figure 1C). With increasing stand age, C:N ratios decreased and subsequently rose in green needles, whereas the reverse was observed in senesced needles (Figure 1D). C:P ratios of both the green and senesced needles tended to increase with stand age, and the slope was greater in senesced needles than in green needles (Figure 1E). Green needle N:P ratios increased and then dropped with stand age, with the maximum being observed in the 42-year-old stands. Senesced needle N:P ratios declined and then increased with stand age, with the minimum being observed in the 22-year-old stands (Figure 1F).

### NuRE and Its Relationships With Needle and Soil Stoichiometry

**Figure 2:**
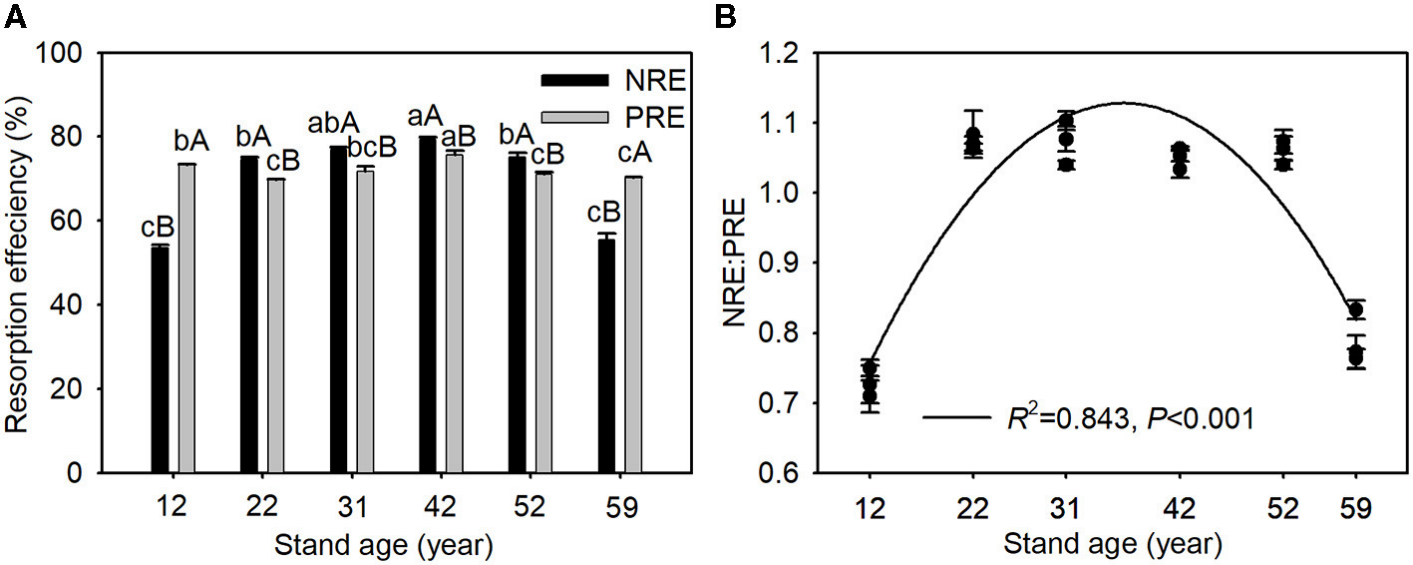
Changes in N and P resorption efficiency **(A)** and NRE:PRE **(B)** along the chronosequence of Mongolian pine plantations. Data are shown as the mean ± SE (*n* = 3). Different lowercases indicate significant differences among stand ages within the same element at *P* < 0.05. Different capital letters indicate significant differences between N and P resorption efficiency within the same stand age at *P* < 0.05.

Nitrogen resorption efficiency, PRE, and NRE:PRE ratios exhibited a trend of initial increase and subsequent decline with stand age. NRE was significantly higher than PRE in the 22-, 31-, 42-, and 52-year-old stands but significantly lower in the 12- and 59-year-old stands (Figure 2). NRE was positively correlated with green needle N concentration and N:P ratio, and senesced needle C:N ratio, but was negatively related to green needle C:N ratio, and senesced needle N concentration and N:P ratio. However, PRE was negatively correlated with senesced needle P concentration, soil-available N concentration, and available N:P ratio (Table 3).

**Table 3:** Pearson correlations between nutrient resorption efficiency and nutrient concentrations and stoichiometric ratios in needles and soil.

**Factor**	**Nutrient and ratio**	**NRE**		**PRE**	
		***R***	***P***	***R***	***P***
Green needle	N	0.810	<0.001		
	C:N	−0.630	0.005		
	N:P	0.787	<0.001		
Senesced needle	N	−0.955	<0.001		
	P			−0.596	0.009
	C:N	0.880	<0.001		
	N:P	−0.888	<0.001		
Soil	Available N			−0.545	0.019
	Available N:P			−0.567	0.014

*NRE and PRE represent N and P resorption efficiency, respectively. R is the Pearson correlation coefficient. The pooled data of all stand ages are used for analyzing the correlations. Only the cases with significance are shown*.

### Stoichiometric Homeostasis

**Figure 3:**
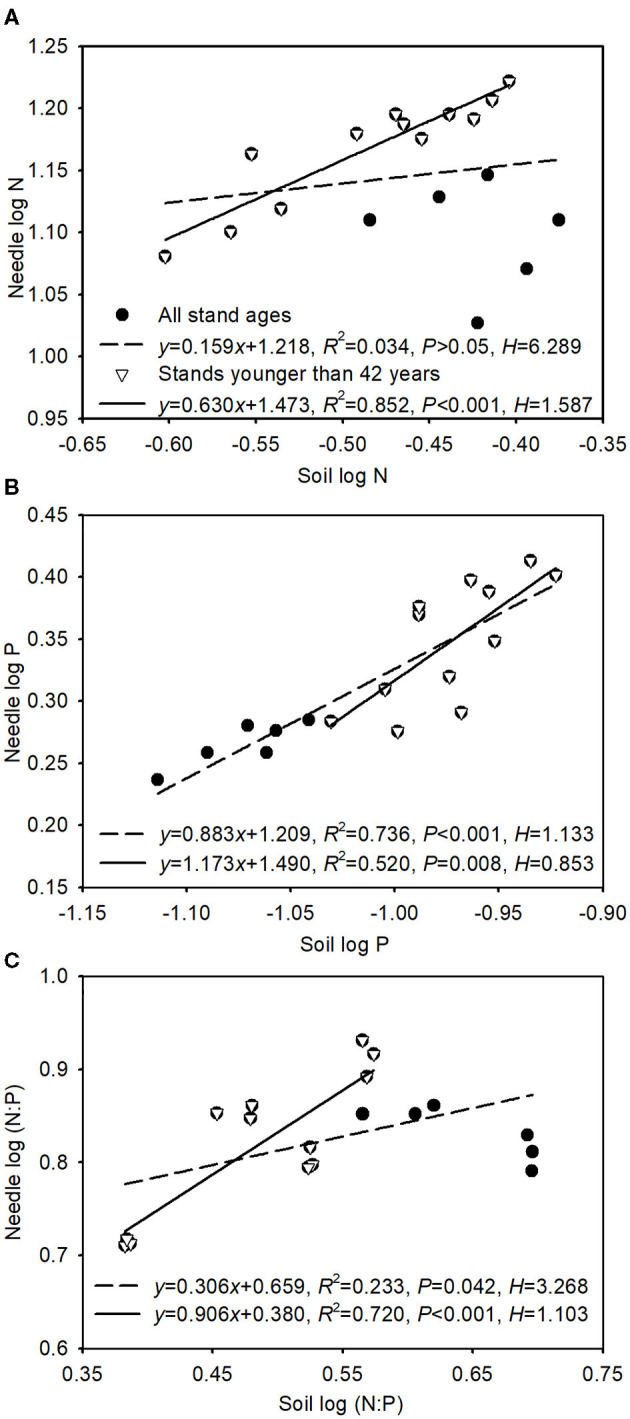
Relationships between green needle and soil for N concentrations **(A)**, P concentrations **(B)**, and N:P **(C)**. Solid and dash lines indicate regression equations for stands younger than 42 years and all stand ages, respectively.

The slope (1/*H*) for N:P ratios between soil and green needle was lower when all stand ages were included than when only stands younger than 42 years were considered (Figure 3). Strict homeostasis was found in N concentrations across all stand ages (Figure 3A). The slopes were between 0 and 1 for N concentrations in stands younger than 42 years, for P concentrations across all stand ages, and for N:P ratios. However, the slope was above 1 for P concentrations in stands younger than 42 years (Figure 3B).

## Discussion

### Changes in Nutrient Resorption Efficiency and Nutrient Limitation

A higher growth potential usually requires more nutrients to support biomass production; therefore, plants should have higher NuRE during their rapid growth phase (Nambiar and Fife, 1991). In disagreement with our first hypothesis, NRE and PRE first increased significantly and then decreased along the chronosequence of the Mongolian pine plantations (Figure 2A), which may be explained by growth variation in Mongolian pine. Mean height and DBH of Mongolian pine increased quickly before the age of 42 years, but slowly after the age of 42 years (Table 1). Meanwhile, increments in the total, aboveground, stem, and root biomass of the Mongolian pine gradually increased until ~40 years of age and then decreased slightly (Zhang et al., 2019). Similar patterns have been reported for *Robinia pseudoacacia* (Deng et al., 2019) and *Pinus massoniana* (Liu et al., 2016) of different ages. NRE and PRE increased with stand development up to 42 years of age, indicating that nutrient resorption was beneficial for internal N and P conservation and reuse to promote growth. However, NRE and PRE decreased with stand development after the age of 42 years, implying that N and P resorption may play a weaker role in conserving nutrients in Mongolian pine plantation stands older than 42 years. A decline in needle biomass and dieback in Mongolian pine plantations often emerges in the plots at stand ages >42 years (our personal observation), which may be explained by possible nutrient deficiencies due to declining nutrient conservation in needles. Although there were significant differences in soil moisture among different stand ages (Table 1; Wang et al., 2021a), no significant relationships were found between NuRE and soil moisture (Supplementary Figure 2).

In this study, we found that soil organic C and total N concentrations in topsoil (depth of 0–10 cm) increased along a chronosequence of Mongolian pine plantations (Table 2), which is in agreement with Li et al. (2012), who reported that Mongolian pine plantations enhanced soil C and N concentrations in the Horqin Sandy Land. Similar results have also been found in *Larix kaempferi* (Yan et al., 2018) and *Populus tremuloides* (Yuan and Chen, 2010) plantations. In contrast to C and N concentrations, soil total P concentrations decreased with stand age (Table 2), with similar results observed for *L. kaempferi* (Yan et al., 2018) and *Metasequoia glyptostroboides* plantations (Zhang et al., 2018). The different dynamics of soil C, N, and P concentrations across the age chronosequence may reflect differences in soil nutrient sources and transformation processes (Kuznetsova et al., 2011). The accumulation of soil C and N is driven mainly by biological factors (e.g., decomposition of plant litter and dead roots), whereas P transformation in soil is driven primarily by biochemical mineralization (e.g., phosphate decomposition, which takes a long time; Deng et al., 2019). With increasing stand age, the plant litter and dead roots in Mongolian pine plantations gradually accumulated (Zhang et al., 2019). Soil water content and holding capacity, soil microorganism quantity, and enzyme activity improved after afforestation (Yang et al., 2005; Li et al., 2012), which may be beneficial for litter decomposition and transformation from large organic residues toward smaller molecular size. Thus, soil C and N concentrations gradually were enhanced with stand development. However, soil P is released as a result of weathering of rocks, which is a very slow process (Vitousek et al., 2010). Increased soil C:P and N:P ratios with stand age (Table 2) have a negative effect on soil P availability (Li et al., 2016), which leads to a decline in soil-available P concentrations along the chronosequence. Furthermore, increased soil-available N:P ratios with stand age (Table 2) may improve the susceptibility to P limitation. These findings indicate that soil P deficiency may occur with the stand development of Mongolian pine plantations.

Green and senesced needle C concentrations increased significantly with stand age (Figure 1A), which may be due to the accumulation of C-rich structural compounds in the older trees compared with the younger trees. A higher C concentration often leads to lower specific leaf area, photosynthetic rates, and growth rates but stronger defensive ability (Niklas and Cobb, 2005), implying slower growth but higher resistance with stand development. Green needle N concentrations exhibited a pattern of initial increase and subsequent decrease with stand age (Figure 1B), which may be related to nutrient requirements of growth along the chronosequence of the Mongolian pine plantations (i.e., needle N concentrations increased as growth accelerated but decreased as growth slowed). P concentrations decreased and C:P ratios increased in both green and senesced needles with increasing stand age (Figures 1C,E), and N:P ratios in senesced needles increased in the older stands (Figure 1F), implying that less P remained in the litter and returned to the soil with stand development, which would reduce the release of P from litter decomposition and negatively affect soil P availability (See et al., 2015). Thus, the continual decline in soil total and available P with increasing stand age was expected (Table 2). Furthermore, senesced needle N concentrations increased greatly while P concentrations decreased slightly with stand development after the age of 42 years (Figures 1B,C), which indicates a minor decrease in P use efficiency but a great decrease in N use efficiency in the trees. Reed et al. (2012) found that plants in comparatively N-rich and P-poor areas should be under selective pressure to use P more efficiently relative to N. Thus, P deficiency may occur in older Mongolian pine plantation stands.

Soil nutrient limitation would result in greater NuRE for plants (Kobe et al., 2005). The greater NRE than PRE in Mongolian pine plantations at most stand ages (Figure 2A) indicates that Mongolian pine stands tended toward N deficiency. Previous studies demonstrated that younger stands were more susceptible to N limitation (Chen et al., 2004), whereas soil P deficiency was enhanced with increasing stand age (Zhao et al., 2009). The rapid growth of young trees requires more P for the production of genetic material (Deng et al., 2019), which may account for the greater PRE and lower NRE:PRE ratios in the 12-year-old stands (Figure 2). However, a declining trend in NRE:PRE ratios was observed from 22 to 59 years old (Figure 2B), indicating that relatively more P was resorbed with increasing stand age. Following the relative nutrient resorption hypothesis that plants would resorb proportionally more N or P under N- or P-limited conditions (Reed et al., 2012), our results imply an increase in P limitation with Mongolian pine stand development. Furthermore, the dieback occurs in Mongolian pine plantations after the age of 42 years, wherein trees grow slowly and thereby have a low demand for N and P. Thus, NRE and PRE ratios decreased (Figure 2A) but senesced needle N increased (Figure 1B), perhaps suggesting a decrease in N limitation after the age of 42 years.

In this study, the homeostatic coefficient for N:P (*H*_N:P_) between soil and needle in Mongolian pine plantations was greater across all stand ages than those younger than 42 years (Figure 3C). Based on the homeostatic degree (Julian et al., 2020), Mongolian pine showed weak homeostasis across all stand ages but non-homeostasis when only stands younger than 42 years were considered. A similar result was reported for *M. glyptostroboides* plantations, in which mature trees were observed to have higher *H*_N:P_ than young trees (Wang et al., 2019). Plants with stoichiometric flexibility (i.e., non-homeostasis) function efficiently when the soil nutrients meet their growth needs, and they are not obliged to regulate nutrient composition to save energy or store nutrients (Meunier et al., 2014). We found that the slope was lower than 1 for N concentrations and higher than 1 for P concentrations before the age of 42 years (Figure 3). These results indicate accumulation of N nutrients in the organism but that P accumulates in the organism faster than it increases in the soil (i.e., P sequestration in biomass), implying that the older stands would be relatively more P-limited. With increasing tree age, leaf N, P, and N:P may be more constrained within a certain range to maintain optimal physiological performance (Fonseca et al., 2018); thereby, older plantations may deploy more conservative nutrient use strategies. Mongolian pine exhibits flexible stoichiometry in younger plantations but relatively conservative stoichiometric homeostasis in older plantations, which is an advantageous strategy in adapting to various regimes.

Needle N:P ratios varied with soil N:P ratios across all ages, and the slope was lower than 1 (Figure 3C), implying that needles did not regulate nutrient composition to save energy and store nutrients (Meunier et al., 2014). Moreover, nutrients in leaves translocate to branches and roots when excessive amounts of soil nutrients are available, whereas under nutrient deficiency leaf nutrients remain constant *via* translocation from other organs and tissues (Wang et al., 2018; Zhang et al., 2018). N:P ratios in leaves and soil constitute an important basis for determining changes in the nutrient limitation status of forest ecosystems with increasing stand age (Liu et al., 2016; Yan et al., 2018). In this study, a green needle N:P < 14 across all ages (Figure 1F) indicated N limitation for plant growth during stand development (Koerselman and Meuleman, 1996). However, stoichiometric homeostasis results in decreased sensitivity of leaf N:P ratios to nutrient limitation compared with soil N:P ratios, which may be because nutrients in leaves are obtained not only from soil but also from other tissues and organs (Garrish et al., 2010), and nutrient resorption from senescing organs enables plants to conserve and reuse nutrients (Aerts, 1996). Moreover, soil-available nutrients can reflect the ability of the soil to supply plants with nutrients (Yu et al., 2011). We found that soil-available N:P ratios correlated with soil-total N:P ratios but not with needle N:P ratios (Supplementary Figure 3), which indicates that soil N:P ratios may be a better indicator than leaf N:P ratios of the variations in soil nutrient. In addition, other organs (such as branch, root; Wang et al., 2021b) and needle age (Yuan et al., 2017) have great influences on nutrient uptake, transport, consumption, and storage. Further research is needed to consider nutrient allocation and translocation among different organs and age-class needles.

In general, based on the results of variations in soil and needle stoichiometry, NRE:PRE ratios, and stoichiometric homeostasis, we found an increase in P limitation and a decrease in N limitation with Mongolian pine stand development. Strictly speaking, the nutrient limitation can only be determined experimentally (i.e., increase in the rate of an ecosystem process by nutrient addition; Tanner et al., 1998; Camenzind et al., 2018), but results obtained from indirect metrics may offer important information due to the relative absence or difficulty in the direct assessment of nutrient limitation in many biomes (Reed et al., 2012).

### Relationships Between Nutrient Resorption and Stoichiometry and Homeostasis

In accordance with our second hypothesis, NRE was negatively correlated with senesced needle N concentrations and N:P ratios but positively correlated with senesced needle C:N ratios (Table 3). In general, higher litter N concentrations and N:P ratios but lower C:N ratios would improve litter quality and decomposition rates (William et al., 2007), thereby enhancing soil nutrient availability, reducing plant nutrient limitation, and reducing NRE (Yan et al., 2016). Positive feedback between NRE and senesced needle N stoichiometry was found during the development of Mongolian pine plantations, indicating that senesced leaf N stoichiometry was a good indicator for NRE. However, in green needles, NRE was positively correlated with N concentrations and N:P ratios, but negatively correlated with C:N ratios (Table 3), contrary to our second hypothesis. The ratio of soluble to insoluble N in the leaves is related to NRE (Pugnaire and Chapin, 1993). Leaf soluble/mobilizable N is resorbed during senescence, stored in stems and roots, and remobilized to support new growth in the next growing season, whereas insoluble N is sequestrated in cell wall proteins and then removed with leaf abscission (Kobe et al., 2005). These results indicate a greater ratio of soluble to insoluble N in the leaves, which is beneficial for the redistribution and reutilization of N.

Phosphorus resorption efficiency was negatively correlated with senesced needle P concentrations and soil-available N concentrations (Table 3), confirming our second hypothesis. These results indicate that PRE was concurrently affected by multiple elements rather than the cycling of a single element alone (See et al., 2015). The process of P hydrolysis demands greater investments of energy and N in hydrolytic enzymes (Hofmann et al., 2016), which may lead to an increased N uptake from soil. PRE was negatively correlated with the soil-available N:P ratio (Table 3), implying that PRE is lower under lower soil P availability. These findings indicate that PRE may not be the main strategy of Mongolian pine in coping with P-limited conditions; thus, more efficient nutrient absorption by roots becomes important. Nutrient resorption may be an inherent feature of species (Killingbeck, 1996), and genetic differences among plants may be more important in determining NuRE (Luyssaert et al., 2005; Deng et al., 2019).

Maintaining stoichiometric homeostasis is an effective adaptation strategy to cope with changing environments (Sterner and Elser, 2002) and is based on regulating root nutrient capture and/or leaf nutrient resorption (Julian et al., 2020). Although Mongolian pine showed weak homeostasis across stand development, it is non-homeostatic in stands younger than 42 years. This result may be related to nutrient requirements of growth (i.e., trees need more nutrients for growth in stands younger than 42 years, leading to non-homeostasis; however, trees grow slowly and maintain relative homeostasis in stands older than 42 years). The cost for root-nutrient acquisition is progressively higher as soil nutrients get scarcer (Hopmans and Bristow, 2002), and nutrient resorption gets progressively more difficult with low nutrient concentrations in leaves (Wright and Westoby, 2003). Thus, decreased soil-available N and P concentrations (Table 2) and needle N and P concentrations after 42 years of age (Figure 1) may lead to the variations in homeostasis with stand age. Julian et al. (2020) found that nutrient resorption did not vary with growth environments, which supports the notion of weak or non-homeostatic behavior of plants. In this study, no significant differences were found in both NRE among 22-, 31-, 52-year-old stands and in PRE among 22-, 31-, 52- and 59-year-old stands (Figure 2). Thus, the relative constant nutrient resorption in most stands indicates weak or non-homeostasis for Mongolian pine. NRE and PRE decreased after the age of 42 years (Figure 2), whereas foliar N:P homeostasis was lower prior to the age of 42 years than when all stand ages were included (Figure 3). The opposite patterns of change were also found by Peng et al. (2016), who reported that *Amaranthus mangostanus* displayed contrasting nutrient homeostasis and resorption responses to environmental nutrient availability across growth stages. These findings indicate that nutrient resorption may play a lesser role in maintaining stoichiometric homeostasis across the chronosequence of Mongolian pine plantations.

### Implications for Forest Management

Our analysis of NRE:PRE ratios and N:P ratios in needles and soil during the stand development of Mongolian pine plantations in Horqin Sandy Land of China indicates that trees may progress from being primarily N limited to being primarily P limited with growth. The reduced P concentrations and increased C:P ratios in senesced needles with stand age imply that less P is returned to the soil as trees age. Furthermore, a substantial amount of the P sequestered in biomass was removed from the plots by thinning. Positive plant–soil feedback would result in soil P deficiency and the occurrence of P limitation with stand development of Mongolian pine plantations. Similar findings have also been observed in other pure plantations that exhibited enhanced P limitation with stand age, including plantations of *P. massoniana* (Liu et al., 2016), *L. kaempferi* (Yan et al., 2018), *M. glyptostroboides* (Zhang et al., 2018), and *R. pseudoacacia* (Deng et al., 2019). However, P limitation did not occur in some mixed forests (Yan et al., 2016; Luo et al., 2020). This raises the question of whether afforestation with pure plantation causes P limitation, which requires further research encompassing other tree species. Adaptive fertilization management strategies offer an appropriate means of alleviating nutrient limitations in timber forests. N and P fertilization may be particularly important for younger and older stands, respectively. Additionally, selection of appropriate N-fixing species for Mongolian pine is a feasible means of establishing mixed forests in sandy land because the introduction of N-fixing species could improve soil N and P availabilities (Wang et al., 2007), which is beneficial for stimulating soil phosphatase activity and enhancing P cycling rates (Deng et al., 2016) because of the supply of highly decomposable litter.

## Conclusion

NRE and PRE exhibit patterns of initial increase and subsequent decline along the chronosequence of Mongolian pine plantations, which are more dependent on the nutrient requirements of growth than on nutrient limitation. Nutrient resorption plays a lesser role in maintaining stoichiometric homeostasis. Nutrient resorption and stoichiometric homeostasis would contribute to a decrease in N limitation and an increase in P limitation with stand development of Mongolian pine plantations. P limitation often occurs with stand development in pure plantations. Therefore, the establishment of mixed plantations may represent a more promising strategy for afforestation.

## Data Availability Statement

The raw data supporting the conclusions of this article will be made available by the authors, without undue reservation.

## Author Contributions

KW and RZ conceived and designed the study. KW and YL performed the experiments. KW and GW wrote the paper. LS, TY, and GW reviewed and edited the manuscript. All authors read and approved the manuscript.

## Conflict of Interest

The authors declare that the research was conducted in the absence of any commercial or financial relationships that could be construed as a potential conflict of interest.

## Publisher's Note

All claims expressed in this article are solely those of the authors and do not necessarily represent those of their affiliated organizations, or those of the publisher, the editors and the reviewers. Any product that may be evaluated in this article, or claim that may be made by its manufacturer, is not guaranteed or endorsed by the publisher.

## Supplementary Material

Supplementary Figure 1 Introduction regions of the Mongolian pine plantations in the “Three Norths” area of China (northwest, north, and northeast China) and location of the study site.

Supplementary Figure 2 Relationships between soil moisture and NRE **(A)**, and PRE **(B)** across all stand ages.

Supplementary Figure 3 Relationships between soil-available N:P and soil total N:P **(A)**, and needle N:P **(B)** across all stand ages.

## References

[B1] Aerts R. (1996). Nutrient resorption from senescing leaves of perennials: are there general patterns? J. Ecol. 84, 597–608. 10.2307/2261481

[B2] Bao S. D. (2000). Soil and Agriculture Chemistry Analysis. Beijing: China Agriculture Press.

[B3] Brant A. N. Chen H. Y. H. (2015). Patterns and mechanisms of nutrient resorption in plants. Crit. Rev. Plant Sci. 34, 471–486. 10.1080/07352689.2015.1078611

[B4] Camenzind T. Hättenschwiler S. Treseder K. Lehmann A. Rillig M. C. (2018). Nutrient limitation of soil microbial processes in tropical forests. Ecol. Monogr. 88, 4–21. 10.1002/ecm.1279

[B5] Chen F. S. Niklas K. J. Liu Y. Fang X. M. Wan S. Z. Wang H. M. (2015). Nitrogen and phosphorus additions alter nutrient dynamics but not resorption efficiencies of Chinese fir leaves and twigs differing in age. Tree Physiol. 35, 1106–1117. 10.1093/treephys/tpv076 26358049

[B6] Chen G. S. Zeng D. H. Chen F. S. (2004). Concentrations of foliar and surface soil in nutrients *Pinus* spp. plantations in relation to species and stand age in Zhanggutai sandy land, northeast China. J. For. Res. 15, 11–18. 10.1007/BF02858003

[B7] Deng J. Wang S. Ren C. J. Zhang W. Zhao F. Z. Li X. F.. (2019). Nitrogen and phosphorus resorption in relation to nutrition limitation along the chronosequence of Black Locust (*Robinia pseudoacacia* L.) plantation. Forests 10, 261. 10.3390/f10030261

[B8] Deng M. F. Liu L. L. Sun Z. Z. Piao S. L. Ma Y. C. Chen Y. W.. (2016). Increased phosphate uptake but not resorption alleviates phosphorus deficiency induced by nitrogen deposition in temperate *Larix principisrupprechtii* plantations. New Phytol. 212, 1019–1029. 10.1111/nph.14083 27400237

[B9] Elser J. Fagan W. Kerkhoff A. Swenson N. Enquist B. (2010). Biological stoichiometry of plant production: metabolism, scaling and ecological response to global change. New Phytol. 186, 593–608. 10.1111/j.1469-8137.2010.03214.x 20298486

[B10] Fan H. Wu J. Liu W. Yuan Y. Hu L. Cai Q. (2015). Linkages of plant and soil C:N:P stoichiometry and their relationships to forest growth in subtropical plantations. Plant Soil 392, 127–138. 10.1007/s11104-015-2444-2

[B11] Fonseca J. G. Calderan-Rodrigues M. J. de Moraes F. E. Cataldi T. R. Jamet E. Labate C. A. (2018). Cell wall proteome of sugarcane young and mature leaves and stems. Proteomics 18:1700129. 10.1002/pmic.201700129 29274249

[B12] Food and Agriculture Organization of the United Nations (2015). International Soil Classification System for Naming Soils and Creating Legends for Soil Maps, Update 2015. World Soil Resources Reports. Rome: Food and Agriculture Organization of the United Nations.

[B13] Garrish V. Cernusak L. A. Winter K. Turner B. L. (2010). Nitrogen to phosphorus ratio of plant biomass versus soil solution in a tropical pioneer tree, Ficus insipida. J. Exp. Bot. 61, 3735–3748. 10.1093/jxb/erq183 20591897 PMC2921206

[B14] Gu Q. Zamin T. J. Grogan P. (2017). Stoichiometric homeostasis: a test to predict tundra vascular plant species and community-level response to climate change. Arct. Sci. 3, 320–333. 10.1139/as-2016-0032

[B15] Hofmann K. Heuck C. Spohn M. (2016). Phosphorus resorption by young beech trees and soil phosphatase activity as dependent on phosphorus availability. Oecologia 181, 369–379. 10.1007/s00442-016-3581-x 26875186

[B16] Hopmans J. W. Bristow K. L. (2002). Current capabilities and future needs of root water and nutrient uptake modeling, in Advances in Agronomy, ed Sparks D. L. (Cambridge: Academic Press), 103–183. 10.1016/S0065-2113(02)77014-4

[B17] Julian P. Gerber S. Bhomia R. K. King J. Osborne T. Z. Wright A. L. (2020). Understanding stoichiometric mechanisms of nutrient retention in wetland macrophytes: stoichiometric homeostasis along a nutrient gradient in a subtropical wetland. Oecologia 193, 969–980. 10.1007/s00442-020-04722-9 32725299

[B18] Killingbeck K. T. (1996). Nutrient in senesced leaves: keys to the research for potential resorption and resorption proficiency. Ecology 77, 1716–1727. 10.2307/2265777

[B19] Kobe R. K. Lepczyk C. A. Iyer M. (2005). Resorption efficiency decreases with increasing green leaf nutrients in a global data set. Ecology 86, 2780–2792. 10.1890/04-1830

[B20] Koerselman W. Meuleman A. F. (1996). The vegetation N: P ratio: a new tool to detect the nature of nutrient limitation. J. Appl. Ecol. 33, 1441–1450. 10.2307/2404783

[B21] Kuznetsova T. Lukjanova A. Mandre M. Lõhmus K. (2011). Aboveground biomass and nutrient accumulation dynamics in young black alder, silver birch and Scots pine plantations on reclaimed oil shale mining areas in Estonia. For. Ecol. Manag. 262, 56–64. 10.1016/j.foreco.2010.09.030

[B22] Li Y. Niu S. L. Yu G. R. (2016). Aggravated phosphorus limitation on biomass production under increasing nitrogen loading: a meta-analysis. Glob. Chang. Biol. 22, 934–943. 10.1111/gcb.13125 26463578

[B23] Li Y. L. Chen J. Cui J. Y. Zhao X. Y. Zhang T. H. (2013). Nutrient resorption in Caragana microphylla along a chronosequence of plantations: implications for desertified land restoration in North China. Ecol. Eng. 53, 299–305. 10.1016/j.ecoleng.2012.12.061

[B24] Li Y. Q. Awada T. Zhou X. H. Shang W. Chen Y. P. Zuo X. A.. (2012). Mongolian pine plantations enhance soil physico-chemical properties and carbon and nitrogen capacities in semi-arid degraded sandy land in China. Appl. Soil Ecol. 56, 1–9. 10.1016/j.apsoil.2012.01.007

[B25] Liu J. T. Gu Z. J. Shao H. B. Zhou F. Peng S. Y. (2016). N-P stoichiometry in soil and leaves of *Pinus massoniana* forest at different stand ages in the subtropical soil erosion area of China. Environ. Earth Sci. 75:1091. 10.1007/s12665-016-5888-7

[B26] Luo X. Z. Hou E. Q. Chen J. Q. Li J. Zhang L. L. Zang X. W.. (2020). Dynamics of carbon, nitrogen, and phosphorus stocks and stoichiometry resulting from conversion of primary broadleaf forest to plantation and secondary forest in subtropical China. Catena 193:104606. 10.1016/j.catena.2020.104606

[B27] Luyssaert S. Staelens J. De Schrijver A. (2005). Does the commonly used estimator of nutrient resorption in tree foliage actually measure what it claims to? Oecologia 144, 177–186. 10.1007/s00442-005-0085-5 15891824

[B28] Meunier C. L. Malzahn A. M. Boersma M. (2014). A new approach to homeostatic regulation: towards a unified view of physiological and ecological concepts. PLoS ONE 9:e107737. 10.1371/journal.pone.0107737 25247989 PMC4172659

[B29] Nambiar E. K. S. Fife D. N. (1991). Nutrient retranslocation in temperate conifers. Tree Physiol. 9, 185–207. 10.1093/treephys/9.1-2.185 14972864

[B30] Niklas K. J. Cobb E. D. (2005). N, P, and C stoichiometry of *Eranthis hyemalis* (Ranunculaceae) and the allometry of plant growth. Am. J. Bot. 92, 1256–1263. 10.3732/ajb.92.8.1256 21646146

[B31] Peng H. Y. Chen Y. H. Yan Z. B. Han W. X. (2016). Stage-dependent stoichiometric homeostasis and responses of nutrient resorption in *Amaranthus mangostanus* to nitrogen and phosphorus addition. Sci. Rep. 6:37219. 10.1038/srep37219 27849041 PMC5110967

[B32] Pugnaire F. I. Chapin F. S. (1993). Controls over nutrient resorption from leaves of evergreen mediterranean species. Ecology 74, 124–129. 10.2307/1939507

[B33] Reed S. C. Townsend A. R. Davidson E. A. Cleveland C. C. (2012). Stoichiometric patterns in foliar nutrient resorption across multiple scales. New Phytol. 196, 173–180. 10.1111/j.1469-8137.2012.04249.x 22882279

[B34] See C. R. Yanai R. D. Fisk M. C. Vadeboncoeur M. A. Quintero B. A. Fahey T. J. (2015). Soil nitrogen affects phosphorus recycling: foliar resorption and plant-soil feedbacks in a northern hardwood forest. Ecology 96, 2488–2498. 10.1890/15-0188.1 26594705

[B35] Song L. N. Zhu J. J. Zhang J. X. Zhang T. Wang K. Wang G. C.. (2019). Effect of drought and topographic position on depth of soil water extraction of *Pinus sylvestris* L. var. mongolica Litv. trees in a semiarid sandy region, Northeast China. Forests 10:310. 10.3390/f10050370

[B36] Sterner R. W. Elser J. J. (2002). Ecological Stoichiometry: The Biology of Elements from Molecules to the Biosphere. Princeton, NJ: Princeton University Press. 10.1515/9781400885695

[B37] Tanner E. V. J. Vitousek P. M. Cuevas E. (1998). Experimental investigation of nutrient limitation of forest growth on wet tropical mountains. Ecology 79, 10–22. 10.1890/0012-9658(1998)079[0010:EIONLO]2.0.CO;2

[B38] Tully K. L. Wood T. E. Schwantes A. M. Lawrence D. (2013). Soil nutrient availability and reproductive effort drive patterns in nutrient resorption in Pentaclethra macroloba. Ecology 94, 930–940. 10.1890/12-0781.1

[B39] Vergutz L. Manzoni S. Porporato A. Novais R. F. Jackson R. B. (2012). Global resorption efficiencies and concentrations of carbon and nutrients in leaves of terrestrial plants. Ecol. Monogr. 82, 205–220. 10.1890/11-0416.1

[B40] Vitousek P. M. Porder S. Houlton B. Z. Chadwick O. A. (2010). Terrestrial phosphorus limitation: mechanisms, implications, and nitrogen-phosphorus interactions. Ecol. Appl. 20, 5–15. 10.1890/08-0127.1 20349827

[B41] Wang J. N. Wang J. Y. Wang L. Zhang H. Guo Z. W. Wang G. G.. (2019). Does stoichiometric homeostasis differ among tree organs and with tree age? For. Ecol. Manage. 453, 117637. 10.1016/j.foreco.2019.117637

[B42] Wang J. Y. Wang J. N. Guo W. H. Li Y. G. Wang G. G. Wu T. G. (2018). Stoichiometric homeostasis, physiology, and growth responses of three tree species to nitrogen and phosphorus addition. Trees Struct. Funct. 32, 1377–1386. 10.1007/s00468-018-1719-7

[B43] Wang K. Pang Y. Y. Zhang R. S. Shen C. Song L. N. (2021a). Allocation characteristics of non-structural carbohydrates of *Pinus sylvestris* var. mongolica with different ages. Chin. J. Ecol. 40, 1264–1274. 10.13292/j.1000-4890.202105.022

[B44] Wang K. Song L. N. Lü L. Y. Zhang L. Qin Z. Y. (2014). Fine root biomass vertical distribution character of main afforestation tree species in Horqin Sandy Land. Bull. Bot. Res. 34, 824–828. 10.7525/j.issn.1673-5102.2014.06.018

[B45] Wang K. Zhang R. S. Song L. N. Yan T. Na E. H. (2021b). Comparison of C:N:P stoichiometry in the plant-litter-soil system between poplar and elm plantations in the Horqin Sandy Land, China. Front. Plant Sci. 12:655517. 10.3389/fpls.2021.655517 33981324 PMC8107480

[B46] Wang Y. P. Houlton B. Z. Field C. B. (2007). A model of biogeochemical cycles of carbon, nitrogen, and phosphorus including symbiotic nitrogen fixation and phosphatase production. *Glob. Biogeochem*. Cycles 21:GB1018. 10.1029/2006GB002797

[B47] William P. Silver W. L. Burke I. C. Leo G. Harmon M. E. Currie W. S.. (2007). Global-scale similarities in nitrogen release patterns during long-term decomposition. Science 315, 361–364. 10.1126/science.1134853 17234944

[B48] Wright I. J. Westoby M. (2003). Nutrient concentration, resorption and lifespan: leaf traits of Australian sclerophyll species. Funct. Ecol. 17, 10–19. 10.1046/j.1365-2435.2003.00694.x

[B49] Yan T. Lü X. T. Yang K. Zhu J. J. (2016). Leaf nutrient dynamics and nutrient resorption: a comparison between larch plantations and adjacent secondary forests in Northeast China. J. Plant Ecol. 9, 165–173. 10.1093/jpe/rtv034

[B50] Yan T. Lü X. T. Zhu J. J. Yang K. Yu L. Z. Gao T. (2018). Changes in nitrogen and phosphorus cycling suggest a transition to phosphorus limitation with the stand development of larch plantations. Plant Soil 422, 385–396. 10.1007/s11104-017-3473-9

[B51] Yang T. Xu H. Li H. Fang D. H. Zhu J. J. (2005). Soil nutrient, microorganism and enzyme activity in *Pinus sylvestris* plantations. J. Soil Water Conser. 19, 50–53. 10.13870/j.cnki.stbcxb.2005.03.013

[B52] Ye G. F. Zhang S. J. Zhang L. H. Lin Y. M. Wei S. D. Liao M. M.. (2012). Age related changes in nutrient resorption patterns and tannin concentration of *Casuarina equisetifolia* plantations. J. Trop. For. Sci. 24, 546–556. 10.3832/ifor0627-005

[B53] Yu Q. Elser J. J. He N. P. Wu H. H. Chen Q. S. Zhang G. M.. (2011). Stoichiometric homeostasis of vascular plants in the Inner Mongolia grassland. Oecologia 166, 1–10. 10.1007/s00442-010-1902-z 21221646

[B54] Yuan Z. Y. Chen H. Y. H. (2010). Changes in nitrogen resorption of trembling aspen (*Populus tremuloides*) with stand development. Plant Soil 327, 121–129. 10.1007/s11104-009-0036-8

[B55] Yuan Z. Y. Shi X. R. Jiao F. Han F. P. (2017). N and P resorption as functions of the needle age class in two conifer trees. J. Plant Ecol. 11, 780–788. 10.1093/jpe/rtx055

[B56] Zhang H. Wang J. N. Wang J. Y. Guo Z. W. Wang G. G. Zeng D. H.. (2018). Tree stoichiometry and nutrient resorption along a chronosequence of *Metasequoia glyptostroboides* forests in coastal China. For. Ecol. Manage. 430, 445–450. 10.1016/j.foreco.2018.08.037

[B57] Zhang X. Zhang X. L. Han H. Shi Z. J. Yang X. H. (2019). Biomass accumulation and carbon sequestration in an age-sequence of Mongolian pine plantations in Horqin Sandy Land, China. Forests 10:197. 10.3390/f10020197

[B58] Zhang Y. G. Xu Z. W. Jiang D. M. Jiang Y. (2013). Soil exchangeable base cations along a chronosequence of Caragana microphylla plantation in a semi-arid sandy land, China. J. Arid Land 5, 42–50. 10.1007/s40333-013-0140-8

[B59] Zhao Q. Zeng D. H. Fan Z. P. Yu Z. Y. Hu Y. L. Zhang J. W. (2009). Seasonal variations in phosphorus fractions in semiarid sandy soils under different vegetation types. For. Ecol. Manage. 258, 1376–1382. 10.1016/j.foreco.2009.06.047

[B60] Zhu J. J. Tan H. Kang H. Z. Xu M. L. (2006). Comparison of foliar nutrient concentrations between natural and artificial forests of *Pinus sylvestris* var. mongolica on sandy land, China. J. For. Res. 17, 177–184. 10.1007/s11676-006-0042-0

