# Supplementary figures and images for: Linkages Between Nutrient Resorption and Ecological Stoichiometry and Homeostasis Along a Chronosequence of Mongolian Pine Plantations
Source: Front Plant Sci. 2021 Aug 13;12:692683. doi: 10.3389/fpls.2021.692683 (PMC8414255; doi:10.3389/fpls.2021.692683)

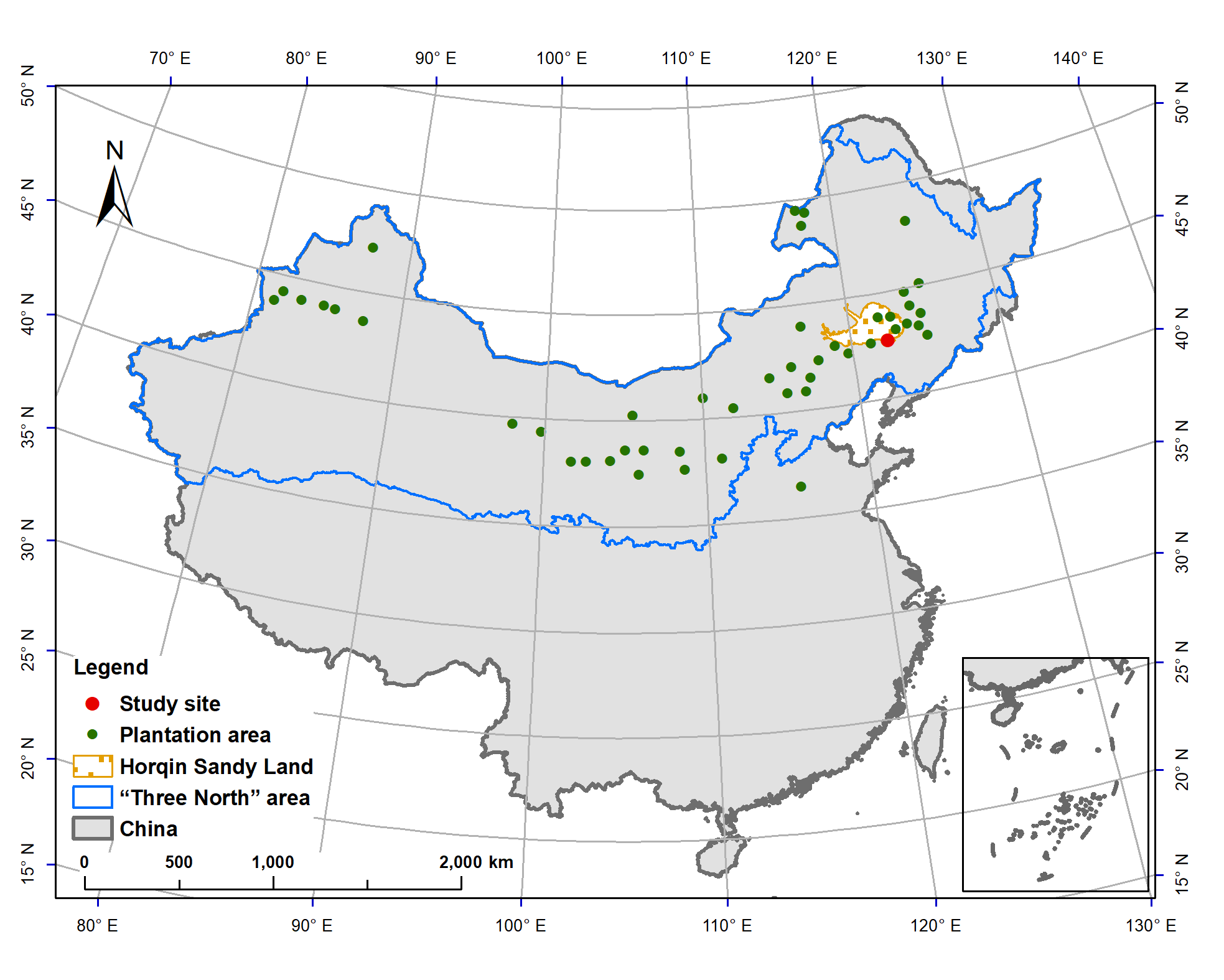

Supplement: Supplementary Figure 1 — Introduction regions of the Mongolian pine plantations in the “Three Norths” area of China (northwest, north, and northeast China) and location of the study site. [file Image_1.TIF]

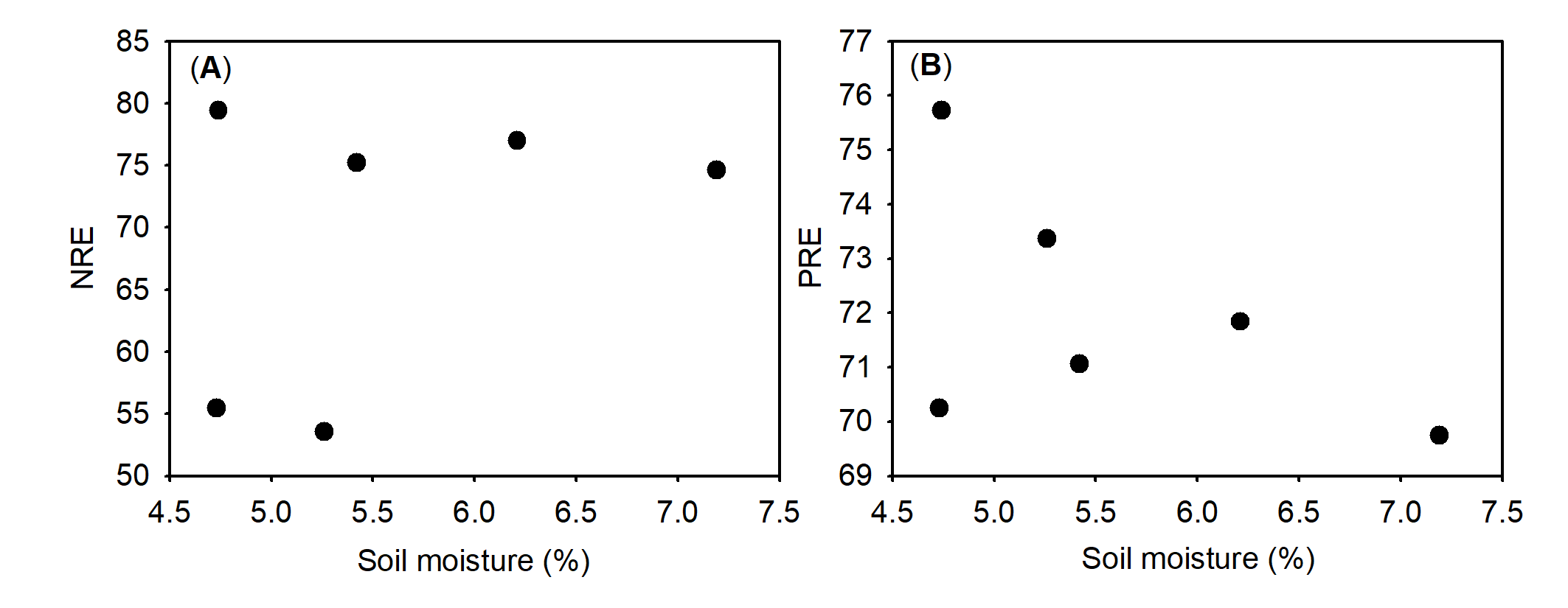

Supplement: Supplementary Figure 2 — Relationships between soil moisture and NRE (A), and PRE (B) across all stand ages. [file Image_2.TIF]

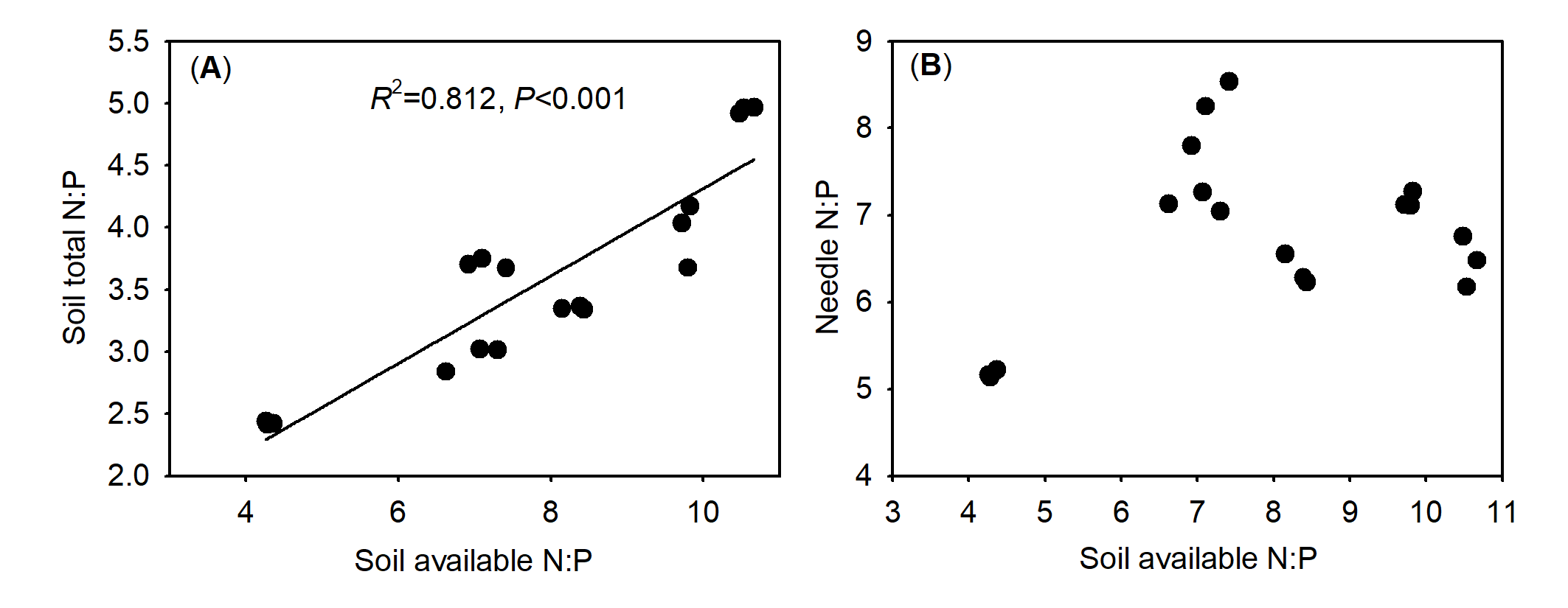

Supplement: Supplementary Figure 3 — Relationships between soil-available N:P and soil total N:P (A), and needle N:P (B) across all stand ages. [file Image_3.TIF]
